# SAHA Overcomes 5-FU Resistance in IFIT2-Depleted Oral Squamous Cell Carcinoma Cells

**DOI:** 10.3390/cancers12123527

**Published:** 2020-11-26

**Authors:** Prabha Regmi, Kuo-Chu Lai, Chung-Ji Liu, Te-Chang Lee

**Affiliations:** 1 Taiwan International Graduate Program in Molecular Medicine, National Yang-Ming University and Academia Sinica, Taipei 11529, Taiwan; prabharegmi@ibms.sinica.edu.tw; 2 Institute of Biomedical Sciences, Academia Sinica, Taipei 11529, Taiwan; 3 Department of Physiology and Pharmacology, College of Medicine, Chang Gung University, Taoyuan City 33302, Taiwan; kuochu@mail.cgu.edu.tw; 4 Department of Oral and Maxillofacial Surgery, Mackay Memorial Hospital, Taipei 10421, Taiwan; cjliu@mmh.org.tw

**Keywords:** 5-FU, IFIT2, SAHA, drug resistance, oral squamous cell carcinoma

## Abstract

**Simple Summary:**

IFIT2 depletion is associated with increased epithelial-mesenchymal transition and metastasis. The main aim of our study was to understand the link between drug resistance and IFIT2 depletion. In this study, we confirmed resistance to multiple common therapeutic drugs, particularly 5-FU, which showed especially high resistance in IFIT2-depleted cells. Interestingly, combination of SAHA and 5-FU overcame 5-FU resistance in IFIT2-depleted cells. Hence, our findings suggest that IFIT2 expression may be used as a biomarker to decide whether to undergo 5-FU treatment, but also the SAHA and 5-FU combination may be a potential new treatment regimen to augment 5-FU therapy in patients with thymidylate synthase-mediated drug-resistant oral squamous cell carcinoma.

**Abstract:**

Interferon-induced protein with tetratricopeptide repeats 2 (IFIT2) is a member of the interferon-stimulated gene family that contains tetratricopeptide repeats (TPRs), which mediate protein–protein interactions in various biological systems. We previously showed the depletion of IFIT2 enhanced cell migration and metastatic activity in oral squamous cell carcinoma (OSCC) cells via the activation of atypical PKC signaling. In this study, we found that IFIT2-knockdown cells displayed higher resistance to 5-fluorouracil (5-FU) than control cells. The comet assay and annexin V analysis showed decreased DNA damage and cell death in IFIT2-knockdown cells compared to control cells treated with 5-FU. Cell cycle progression was also perturbed by 5-FU treatment, with the accumulation of IFIT2-depleted cells in S phase in a time-dependent manner. We further observed the overexpression of thymidylate synthase (TS) and thymidine kinase (TK) in IFIT2-knockdown cells. Inhibition of TS alone or double inhibition of TS and TK1 using the siRNA technique increased susceptibility to 5-FU in IFIT2-knockdown cells. We further identified that suberanilohydroxamic acid (SAHA) treatment decreased the expression of TS in IFIT2-knockdown cells and demonstrated that pretreatment with SAHA sensitized IFIT2-knockdown cells to 5-FU in vitro and in vivo. In conclusion, IFIT2 knockdown enhances TS expression, which mediates 5-FU resistance, and SAHA pretreatment suppresses TS expression and hence sensitizes cells to 5-FU. SAHA will be an effective strategy for the treatment of OSCC patients with 5-FU resistance.

## 1. Introduction

Interferon-induced protein with tetratricopeptide repeats (IFITs) are members of the IFN-stimulated gene family and are considered highly responsive to IFNs, viruses and pathogen-associated molecular patterns [1,2]. The human IFIT family is encoded by four evolutionarily conserved genes, *IFIT1*, *IFIT2*, *IFIT3* and *IFIT5*, which are clustered on chromosome 10 [3]. The IFIT family displays broad-spectrum antiviral functions [4]. However, emerging evidence has also shown that IFIT proteins participate in diverse signaling mechanisms involved in cancer progression and metastasis [5].

Intriguingly, IFIT1, IFIT3 and IFIT5 proteins play significant roles in malignant progression [2,6,7], whereas numerous reports have shown that decreased IFIT2 expression is associated with tumor progression and poor survival of patients with different cancers [8,9]. Overexpression of the IFIT2 protein was found to inhibit proliferation or promote apoptosis in a variety of cancer cell lines [10,11,12,13,14,15]. Our previous studies demonstrated that IFIT2 interacted with the cytoskeleton and that its depletion induced epithelial-mesenchymal transition (EMT) in oral squamous cell carcinoma (OSCC) cells [16]. We also showed that IFIT2 depletion activated atypical protein kinase (aPKC) signaling, increased migration, invasion, and metastasis and regulated TNF-α secretion, leading to angiogenesis and chemoresistance in OSCC [17,18]. Therefore, IFIT2 expression may be considered a prognostic biomarker for OSCC.

Oral cancer is the 6th most common cancer globally, and its occurrence varies depending on the demographic region [19]. Among oral cancers, 95% are OSCC and possess a highly malignant phenotype that results in cervical nodal and distant metastases [20,21]. Loco-regional recurrence, metastasis and drug resistance are the major causes of mortality in OSCC patients [22]. Surgical resection is the primary approach in the clinic along with adjuvant radiation or chemotherapy in advanced stages [23]. Unfortunately, the five-year overall survival rate has remained at 50–60% for decades despite advancements in diagnosis and treatment [24]. The treatment plan and prognostic evaluations are determined after understanding the biological and clinical behavior of tumors in reliant to American Joint Committee on Cancer (AJCC)/The International Union Against Cancer(UICC)staging system or conventional WHO guidelines that fails to address the robust prognostic stratification [25,26]. Therefore, prognosis in OSCC is very poor because it is influenced by multiple factors such as TNM staging, perinuclear and lymphovascular tissue invasion, depth of invasion, tumor thickness, size, cell differentiation, biological tumor markers, tumor site, neutrophil to lymphocyte ratio (NLR), tumor-infiltrating lymphocytes (TILs), extracapsular spread (ECS)/extranodal extension (ENE) [25,27,28,29,30]. The heterogeneity of cancer cells within the oral cavity and controversies on staging and grading have given poor prognosis [27]. There is an urgent need to identify reliable prognostic biomarkers in order to develop novel therapeutic approaches to improve the clinical outcomes and survival of patients with OSCC [31,32,33].

The current standard regimen of chemotherapy for recurrent or metastatic OSCC patients is 5-fluorouracil (5-FU), cisplatin, docetaxel and a multiagent combination with a platinum compound and 5-FU [34,35]. The clinical trial on multidrug combinations or monoclonal antibodies targeting EGFR combined with cisplatin and 5-FU improved the median survival but at costs of high toxicity and ultimately resistance [36,37,38,39]. Inhibitors targeting EGFR, angiogenesis, mTOR, PI3K, the proteasome, histone deacetylases (HDACs) and Toll-like receptor antagonists are other clinical trial drugs that have shown discouraging results [40]. Therefore, chemotherapeutic agents remain the first-line drug for the treatment of patients with OSCC.

Metastasis and drug resistance are the main causes of mortality in cancer patients, and various studies have illustrated that EMT is an important bridge between them [41,42,43]. Since IFIT2 depletion is accompanied by EMT [16,17], IFIT2 may modulate drug resistance in OSCC cells. However, the underlying mechanism by which IFIT2 depletion and chemoresistance are associated is largely unknown. Exploration of the role of IFIT2 in chemoresistance may provide valuable information to propose strategies to block cancer progression and metastasis and identify the correct regimen for treatment. In the present study, we investigated the cytotoxicity of various therapeutic drugs and demonstrated that IFIT2-depleted OSCC cells show resistance to 5-FU. In addition, we demonstrated increased thymidylate synthase (TS) expression in IFIT2-depleted cells, whose silencing sensitized OSCC cells to 5-FU. Furthermore, we found that the HDAC inhibitor suberanilohydroxamic acid (SAHA) attenuated 5-FU resistance in IFIT2-depleted cells.

## 2. Results

### 2.1. 5-FU Resistance in IFIT2-Knockdown Cells

**Figure 1:**
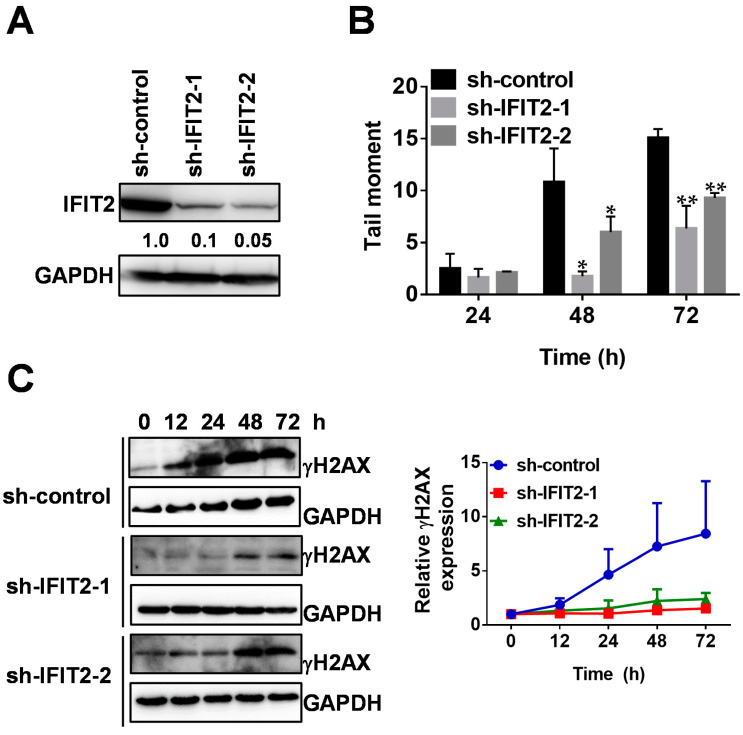
DNA damage in IFIT2-knockdown cells after 5-FU treatment. (**A**) IFIT2 expression in IFIT2-knockdown cells. The IFIT2 protein levels in sh-control, sh-IFIT2-1 and sh-IFIT2-2 cells were determined by Western blotting. GAPDH was used as a loading control. (**B**) Comet assay. Quantitative data from the comet assay. Data are presented as the mean ± SEM of three independent experiments. * *p* < 0.05, ** *p* < 0.01 compared with sh-control cells. (**C**) Induction of γH2AX. Cells were treated with 5-FU for 12, 24, 48 and 72 h. The appearance of γH2AX was determined by Western blotting. The right panel is the quantitative data of the of relative γH2AX expression from three independent experiments.

To evaluate how IFIT2-knockdown cells respond to therapeutic drugs, we treated sh-IFIT2-1 and sh-IFIT2-2 cells (stable IFIT2-knockdown cells) with a number of clinically used therapeutic drugs and performed cytotoxicity assays. The low expression of IFIT2 in these cells was confirmed by Western blotting (Figure 1A). As summarized in Table 1, sh-IFIT2-1 and sh-IFIT2-2 cells were almost four-fold more resistant to 5-FU and 1.2- to 1.7-fold more resistant to other antimetabolites, such as cytarabine, gemcitabine and raltitrexed, compared to sh-control cells. Although IFIT2-knockdown cells did not show significant resistance to topoisomerase I and II inhibitors, such as irinotecan, etoposide and mitoxantrone, sh-IFIT2-1 cells were slightly but significantly resistant to doxorubicin. Although IFIT2-knockdown cells showed 1.4- to 1.7-fold more resistance to oxaliplatin, there were no alterations in sensitivity to other platinum-based drugs, such as cisplatin and carboplatin. Intriguingly, IFIT2-knockdown cells were 1.6- to 1.9-fold more resistant to gefitinib, a tyrosine kinase inhibitor. These results revealed that the depletion of IFIT2 expression indeed modulated cellular sensitivity to antimetabolites, which are usually first-line drugs for the treatment of head and neck cancers in the clinic [44]. In particular, IFIT2-knockdown OSCC cells showed high resistance to 5-FU.

### 2.2. Attenuated DNA Damage Activity of 5-FU in IFIT2-Knockdown Cells

The primary role of 5-FU is to induce DNA damage and trigger cell death; hence, we performed a comet assay to analyze its DNA damage activity in IFIT2-knockdown cells. As shown in Figure S1, we observed significantly longer tail moments in sh-control cells treated with 5-FU than in IFIT2-knockdown cells in a time-dependent manner. The quantitative data are summarized in Figure 1B, in which 5-FU treatment for 48 and 72 h induced lower levels of tail moments in sh-IFIT2-1 and sh-IFIT2-2 cells than in sh-control cells, indicating that 5-FU induced lower levels of DNA damage in IFIT2-knockdown cells than in sh-control cells. We further confirmed the levels of DNA damage induced by 5-FU by detecting the DNA damage marker γH2AX. As shown in Figure 1C, γH2AX levels were significantly increased in a time-dependent manner in sh-control cells, sh-IFIT2-1 cells and sh-IFIT2-2 cells. However, the extent of γH2AX appearance, either time or amount, was lower in sh-IFIT2-1 and sh-IFIT2-2 cells than in sh-control cells. These results revealed that IFIT2 depletion attenuated the DNA damaging activity of 5-FU.

### 2.3. Effect of 5-FU on Cell Cycle Progression and Apoptosis in IFIT2-Knockdown Cells

**Figure 2:**
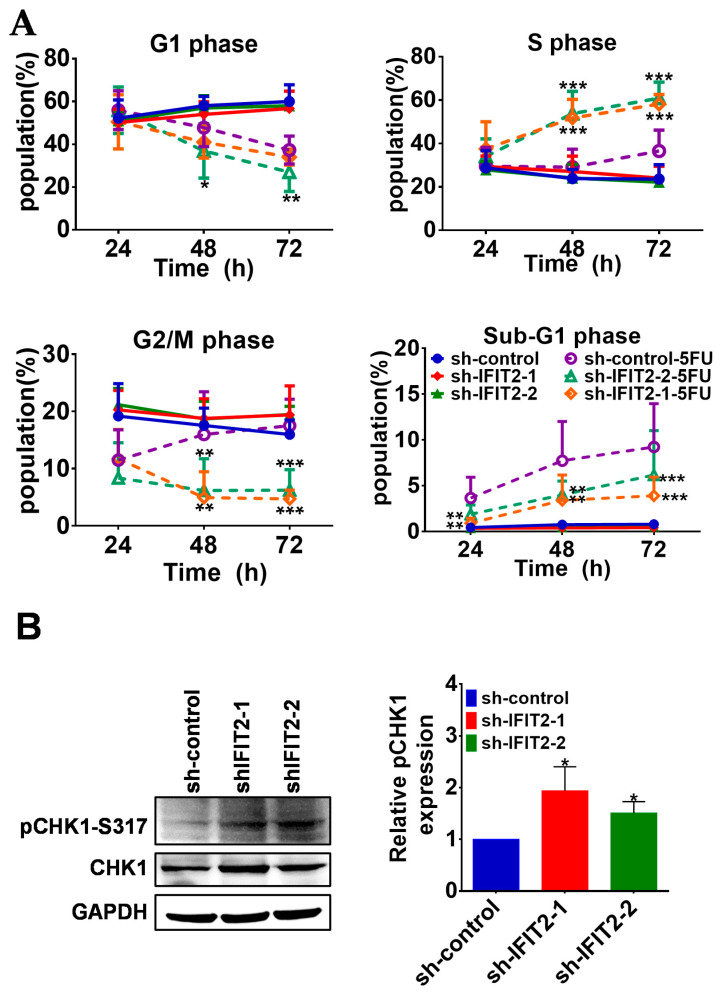
Cell cycle interference by 5-FU. (**A**) Cell cycle analysis. Growing sh-control, sh-IFIT2-1 and sh-IFIT2-2 cells were treated with 10 µM 5-FU for 24, 48 and 72 h. The cells were harvested, and the cell cycle phase was analyzed by flow cytometry as described in the Materials and Methods. The symbols *, ** and *** denote statistically significant differences compared to sh-control at *p* < 0.05, *p* < 0.01 and *p* < 0.001, respectively. (**B**) Increased basal levels of pChk1-S317 in IFIT2-knockdown cells. The lysates of sh-control, sh-IFIT2-1 and sh-IFIT2-2 cells were subjected to Western blot analysis using antibodies against Chk1 and pChK1-S317, respectively. The bar values in the right panel is the quantitative SEM values of relative expression of pCHK1.

Since DNA damaging agents such as 5-FU are known to interfere with cell cycle progression, we therefore investigated the effect of 5-FU on cell cycle progression in IFIT2-knockdown cells. We treated sh-control, sh-IFIT2-1 and sh-IFIT2-2 cells with 10 µM 5-FU for different time periods (24, 48 and 72 h) and determined the cell cycle phase using flow cytometry (Figure S2). As summarized in Figure 2A, we observed that 5-FU treatment decreased the G1 population but slightly increased the S and significantly accumulated the G2/M populations after 48 and 72 h of treatment in sh-control cells. A significant accumulation of the S population (from 30% to 60%) was observed in IFIT2-knockdown cells treated with 5-FU for 48 to 72 h, and subsequently, the G2/M population decreased from 20% in untreated cells to 3–5% after treatment with 5-FU. These results indicated that 5-FU treatment mainly induced the G2/M accumulation in sh-control cells but severely interfered with the progression of the S phase in IFIT2-knockdown cells. Intriguingly, the level of phosphorylated CHK1 (pCHK1-S317) was enhanced in IFIT2-knockdown cells but not in sh-control cells (Figure 2B). Taken together, our results implicated that the DNA damage response was activated in IFIT2-knockdown cells exposed to 5-FU and hence delayed the progression of cells into S phase and G2/M phase.

**Figure 3:**
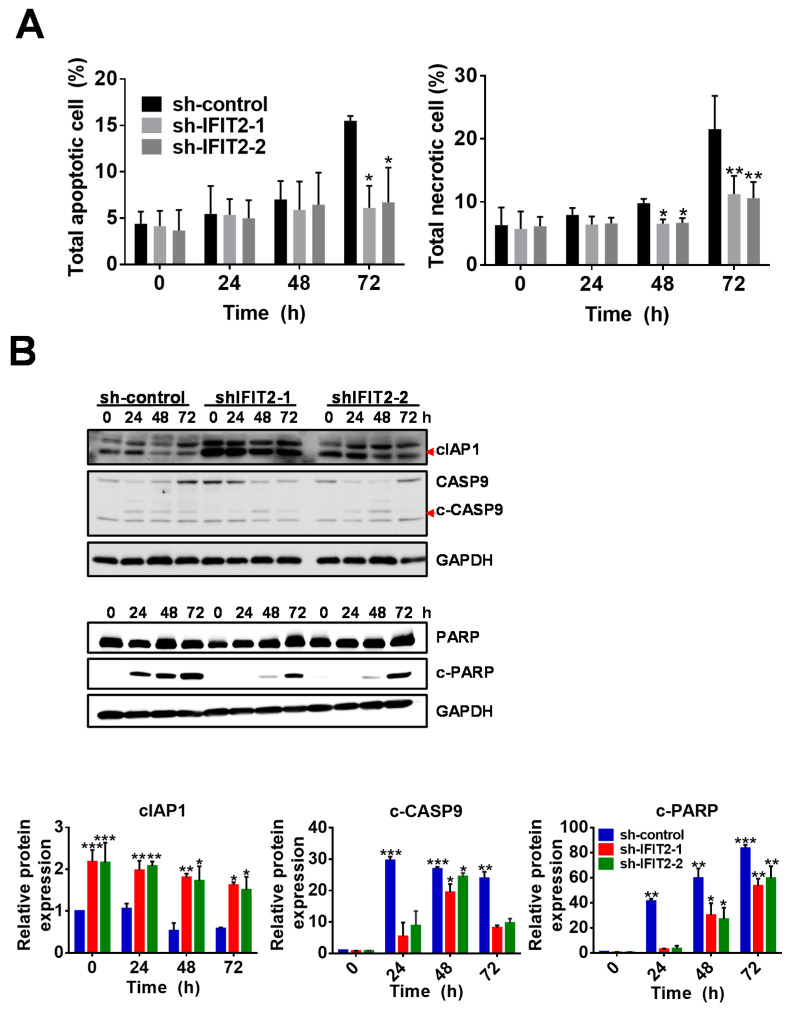
Apoptotic death triggered by 5-FU. (**A**) Quantitative data analysis of apoptotic cell death. Data are presented as the mean ± SEM of three independent experiments. * *p* < 0.05 and ** *p* < 0.01 compared with sh-control cells. (**B**) Expression of the prosurvival protein cIAP1, the protease caspase 9 and cleaved PARP after 5-FU treatment. The cells were treated with 5-FU for 24, 48 and 72 h. The protein levels were determined by Western blotting. GAPDH was used as a loading control. The bar graphs below depict the quantitative densitometry analysis of Western blot data from three independent experiments. The symbols *, ** and *** denote statistically significant differences compared to sh-control at 0 h at *p* < 0.05, *p* < 0.01 and *p* < 0.001, respectively.

Moreover, even though sh-IFIT2-1 and sh-IFIT2-2 cells showed an increase in the subG1 population after treatment for 24 h, sh-control cells showed a significant increase in the subG1 population compared to IFIT2-knockdown cells after 5-FU treatment, implicating that 5-FU induced not only DNA damage but also apoptotic death. We therefore performed an annexin V staining assay to confirm the apoptosis induced by 5-FU treatment. Annexin V staining analysis showed more apoptotic and necrotic sh-control cells than sh-IFIT2-1 and sh-IFIT2-2 cells, and the percentages of sh-control cells that underwent apoptosis and necrosis increased in a time-dependent manner (Figure S3). After sh-control cells were treated with 5-FU for 72 h, the percentages of apoptotic and necrotic cells were 15% and 20% respectively, which were significantly higher than those of IFIT2-knockdown cells (Figure 3A). Immunoblotting was performed to determine caspase-mediated death. Active forms of caspase 9 (c-CASP9) and cleaved PARP (c-PARP) appeared and significantly increased in sh-control cells from 24 to 72 h, whereas c-CASP9 increased only at a peak of 48 h and c-PARP appeared after 48 h and slowly increased at levels lower than sh-control cells (Figure 3B), while the prosurvival marker cIAP1 was expressed at higher levels in knockdown cells than in control cells and showed a slight decrease after 5-FU treatment (Figure 3B).

### 2.4. Altered Expression of 5-FU Metabolic Enzymes in IFIT2-Knockdown Cells

**Figure 4:**
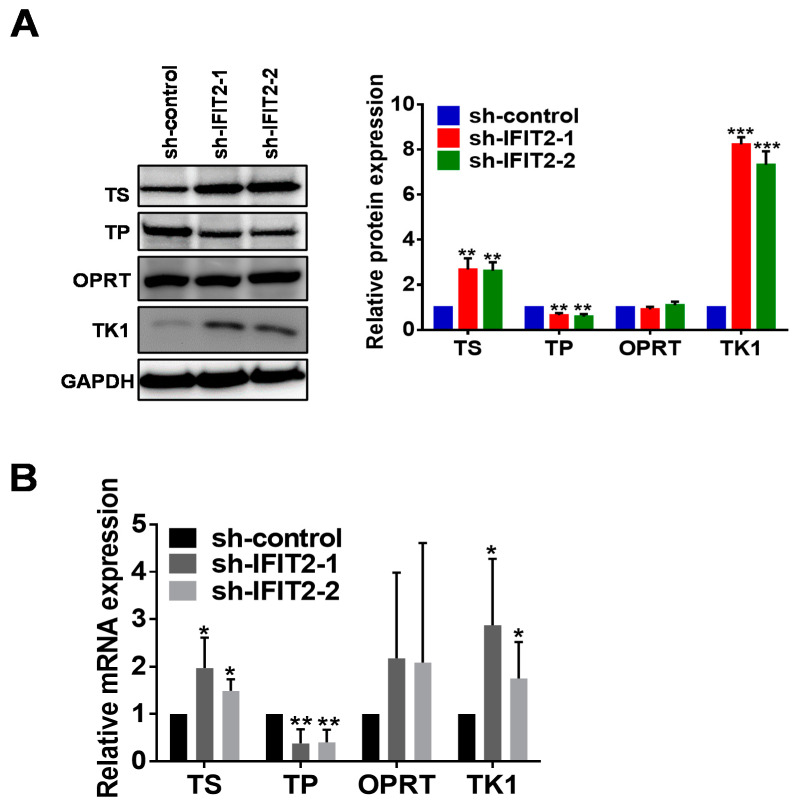

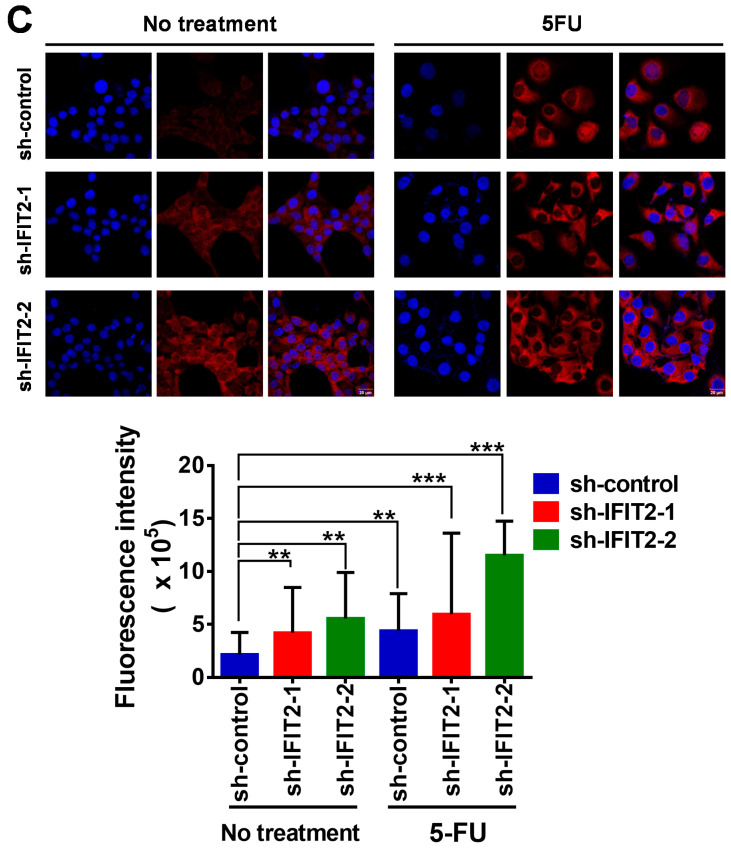

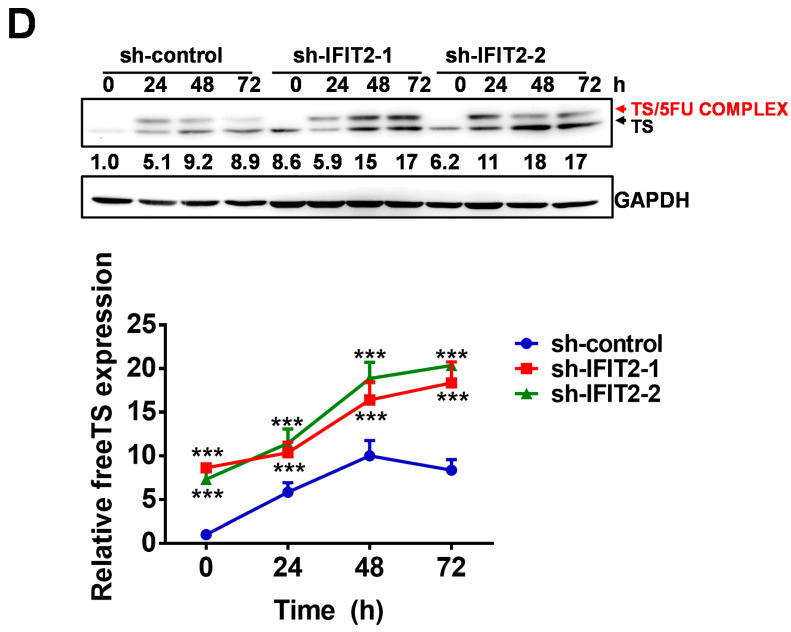
Protein and mRNA levels of 5-FU metabolic enzymes. (**A**) Protein levels. The whole cell lysates of sh-control, sh-IFIT2-1 and sh-IFIT2-1 cells were immunoblotted for TS, TK1, OPRT and TP. Bar graph indicates quantitative densitometry of Western blot analysis of three independent experiments relative to sh-control. The symbols ** and *** denote statistically significant differences compared to sh-control at *p* < 0.01 and *p* < 0.001, respectively. (**B**) mRNA levels. The relative mRNA expression levels of 5-FU metabolic enzymes in sh-control, sh-FIT2-1 and sh-IFIT2-2 cells were determined by Q-PCR. GAPDH mRNA was used as an internal control. * *p* < 0.05, ** *p* < 0.01 denotes a statistically significant difference compared to sh-control. (**C**) Immunostaining. The intracellular distribution of TS in sh-control, sh-FIT2-1 and sh-IFIT2-2 cells with or without 5-FU treatment (10 μM for 48 h) was determined by immunostaining (red). The nuclei were counterstained with DAPI (blue). Quantitative analysis of the mean fluorescence intensity is shown in bar graph. The symbols ** and *** denote statistically significant differences compared to non-treated sh-control at *p* < 0.01 and *p* < 0.001, respectively. (**D**) Formation of the TS/5-FU complex. As described above, the cells were treated with 10 μM 5-FU for various time periods. The upper band (red arrow) denotes the TS/5-FU complex, and the lower band (black arrow) represents free TS. GAPDH was used as a loading control. The numbers represent the relative expression level of Free TS protein in compared to control cells. Quantitative densitometry of Western blot analysis of three independent experiments. *** *p* < 0.001 denotes a statistically significant difference compared to sh-control at 0 h.

We sought to understand 5-FU resistance in IFIT2-knockdown cells, so we performed Q-PCR and Western blot analysis to examine the expression of different enzymes involved in 5-FU metabolism. We observed higher protein expression of TS and thymidine kinase (TK) in IFIT2-knockdown cells than in sh-control cells, but in contrast, the protein levels of thymidine phosphorylase (TP) were lower in IFIT2-knockdown cells than in sh-control cells (Figure 4A). Orotate phosphoribosyl transferase (OPRT) did not show any difference (Figure 4A). Moreover, the relative mRNA expression of TS, TK and TP was consistent with the protein expression (Figure 4B). In addition, we visualized the intracellular localization of TS with or without 5-FU treatment using immunostaining. As expected, TS expression was higher in IFIT2-knockdown cells than in sh-control cells (Figure 4C). However, after 5-FU treatment for 48 h, TS expression was abundantly expressed and localized in both the cytosol and nucleus in control cells, while in IFIT2-knockdown cells, TS still showed predominant localization in the cytosol (Figure 4C). Furthermore, fluorodeoxyuridylate (FdUMP), a major 5-FU metabolite, is considered the main mechanism of action of 5-FU. It inhibits TS by competitively binding with dUMP and forming a covalent ternary complex with TS and 5,10-methylenetetrahydrofolate (CH2-THF) [45]. Therefore, we determined the ternary complex using immunoblotting. As shown in Figure 4D both the TS+FdUMP complex and free TS decreased after 48 h of 5-FU treatment in sh-control cells. However, the TS+FdUMP complex increased by 5-FU treatment and remained constant after 48 h in IFIT-2 depleted cells. Moreover, free TS levels were significantly increased in IFIT2 depleted cells after 48 h and 72 h treatments. The free TS is directly responsible for maintaining the levels of thymidylate and DNA synthesis in the cells after cytotoxic 5-FU treatment, suggesting better coping strategies against 5-FU, thus leading to resistance. Taken together, increased levels of free TS in IFIT2-depleted cells compared to control cells suggest that TS levels are crucial factors for imparting resistance to 5-FU.

**Figure 5:**
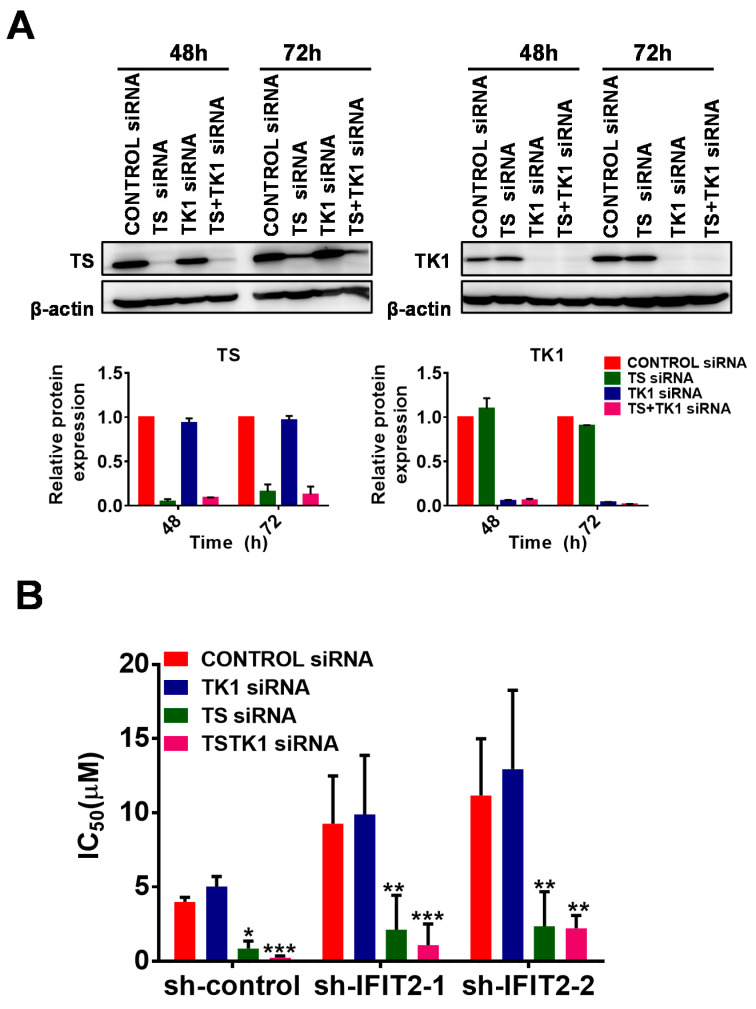
Silencing TS expression sensitizes IFIT2-knockdown cells to 5-FU. (**A**) Silencing TS or TK1 by the siRNA technique. The specific siRNAs for TS and TK1 were transfected into cells either individually or in combination. TS and TK1 expression were confirmed after siRNA transfection in sh-IFIT2-1 cells for 48 h and replating in fresh medium for 72 h. The knockdown efficiency of siRNA transfection measured by detecting the protein levels of TS and TK1 by Western blotting. The quantitative densitometry of Western blot analysis of two independent experiment is shown in the graph. (**B**) IFIT2-knockdown cells were sensitized to 5-FU by silencing TS expression. Transfected siRNAs were seeded into 96-well plates at 3000 cells per well. The cells were treated with 5-FU in a serial dilution from 100 µM for 72 h. At the end of treatment, an aliquot of the Presto blue solution was added, and the fluorescence intensity was measured. IC_50_ values were calculated using CompuSyn software. The symbols *, ** and *** denote statistically significant differences compared to control siRNA at *p* < 0.05, *p* < 0.01 and *p* < 0.001, respectively.

To overcome resistance, a TS siRNA and a TK1 siRNA were separately or jointly transfected into cells, and cytotoxicity was measured. The silencing efficiency of TS and TK1 by the siRNAs is shown in Figure 5A. The values of IC_50_ that decreased by 5-fold in TS-silenced cells and by 5–15-fold in double TS/TK1-silenced cells showed increased sensitivity in TS- and double TS/TK1-silenced sh-IFIT2-1 and sh-IFIT2-2 cells compared to nonsilenced control or TK1-silenced cells (Figure 5B). These results confirmed that enhanced TS levels instead of TK1 is involved in 5-FU resistance in IFIT2 knockdown cells.

### 2.5. Synergistic Inhibition of Tumor Growth by the Combination of SAHA and 5-FU

**Figure 6:**
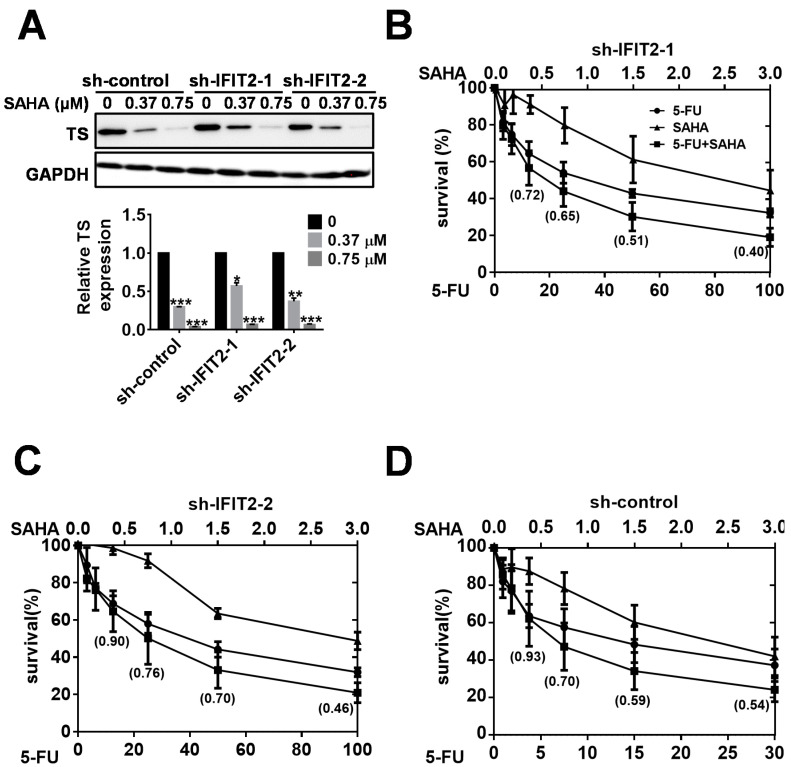
Sensitizing cells to 5-FU with SAHA. (**A**) Reduced expression of TS by SAHA. The cells were treated with SAHA at concentrations of 0, 0.375 and 0.75 μM for 24 h. The protein levels of TS were determined by Western blotting. Bar graph is the quantitative densitometry of Western blot analysis of three independent experiments. The symbols *, ** and *** denote statistically significant differences compared to non-treated cells at *p* < 0.05, *p* < 0.01 and *p* < 0.001, respectively (**B**–**D**). Synergistic cytotoxicity of SAHA and 5-FU. A combination cytotoxicity assay of 5-FU and SAHA was performed at a ratio of 33:1 in sh-IFIT2-1 cells, sh-IFIT2-2 cells and 10:1 in sh-control cells. The CI shown in parentheses was calculated using CompuSyn software, where CI values denote the following: CI = 1, additive effects; CI < 1, synergism; and CI > 1, antagonism.

Our data suggest that TS expression is critical for 5-FU resistance in IFIT2-knockdown cells. To overcome TS-driven 5-FU resistance, we assessed the efficacy of the HDAC inhibitor SAHA. SAHA sensitizes 5-FU-resistant cells in many cancers by suppressing TS expression or increasing the apoptosis pathway [46,47]. We first analyzed its effect on TS protein expression. TS expression was suppressed in a dose-dependent manner after SAHA treatment in all the cell lines (Figure 6A). Then, we performed combination treatment at different dose ratios (5-FU:SAHA; 0.3:1, 0.5:1, 0.8:1, 1:1, 1:7, 2:1, 3:1, 4:1, 7:1, 8:1, 10:1, 13:1, 16:1, 20:1, 27:1, 33:1, 53:1 and 67:1). The synergistic effect was observed with the combination of a low dose of SAHA and a high dose of 5-FU. We identified the optimal ratio of 5-FU and SAHA as 33:1 for the synergistic effect in sh-IFIT-1 and sh-IFIT-2 cells (Figure 6B,C), while 10:1 in sh-control cells (Figure 6D). Combination index (CI) values portrayed the synergism of SAHA and 5-FU at a ratio of 33:1.

**Figure 7:**
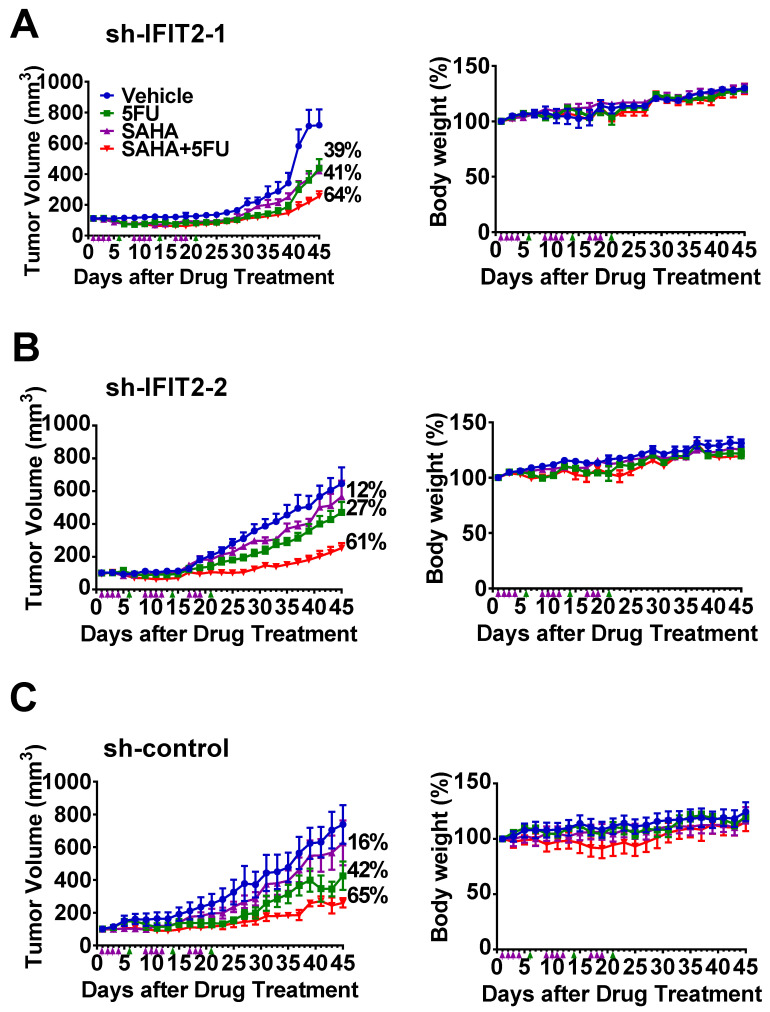
Synergistic inhibition of SAHA and 5-FU in a xenograft tumor model. (**A**–**C**) An aliquot of 10^7^ cells (sh-IFIT2-1, sh-IFIT2-2 and sh-control) was subcutaneously implanted into nude mice. When the tumor reached approximately 100 mm^3^, the mice were randomly divided into four groups (*n* = 4–5 for each group) and treated with vehicle (●), SAHA (▲, 80 mg/kg orally), 5-FU (▲, 100 m/kg i.p.) or a combination of the two drugs (▼). The tumor volume (left) and body weight change (right) of the mice were recorded every other day. The values are shown as the mean ± SD of each group.

To validate the in vitro synergistic inhibition of cell growth in vivo, we further evaluated the efficacy of combined treatment using xenograft models. We compared the effects of 5-FU combined with SAHA with those of SAHA alone, 5-FU alone and vehicle control treatment. In detail, mice were given 80 mg/kg SAHA orally 11 times over 3 weeks and 100 mg/kg 5-FU intraperitonially once a week three times. As shown in Figure 7A,B, in IFIT2-knockdown cells, the 5-FU and SAHA combination resulted in a significant 61–64% reduction in tumor volume, while 5-FU alone resulted in a 27–39% reduction, and SAHA alone resulted in a 12–41% reduction. Similarly, 5-FU and SAHA combination showed tumor suppression of 65%, 5-FU alone 42% and SAHA alone 16% in sh-control cells (Figure 7C). Furthermore, the mice treated with drugs above exhibited no significant loss of body weight (Figure 7A–C). Taken together, our data suggest the synergistic effect of SAHA and 5-FU in overcoming 5-FU resistance.

## 3. Discussion

We previously showed that decreased IFIT2 expression was associated with enhanced migration and metastasis in OSCC cell lines and poor survival in OSCC patients [16,17]. In the present study, we found that among the therapeutic agents with different action modes, IFIT2-knockdown cells conferred resistance primarily to antimetabolites, particularly 5-FU. The 5-FU resistance observed in the current study is consistent with our preliminary studies in which IFIT2-depleted metastatic and xenograft-generated sublines showed resistance to 5-FU by resistance factors of 4.7 and 3.5, respectively [18]. Concerning the mechanistic pathway involved, we identified high TS expression in IFIT2-knockdown cells. Silencing TS expression sensitized IFIT2-knockdown cells to 5-FU. Furthermore, we observed the synergistic effects of the combination of 5-FU and SAHA in suppressing cell growth in culture and decreasing tumor volume in a mouse xenograft model.

Although 5-FU was discovered as an anticancer drug more than 50 years ago, it and its prodrug derivatives are still commonly used for the treatment of a variety of malignancies, including pancreatic, colorectal, breast, and head and neck cancers [48,49,50,51]. It is evident that 5-FU targets cells in S phase, blocks DNA replication, and elicits DNA damage and cell death [52]. Our present results confirmed that the resistance to 5-FU in IFIT2-knockdown cells compared to sh-control cells was associated with shorter 5-FU-induced tail moments (as assessed with comet assays) and γH2AX expression that increased in a time-dependent manner, i.e., decreasing 5-FU-induced DNA damage. Comet assays (alkaline single cell gel electrophoresis) have been widely adopted to detect chemical-induced DNA breaks [53]. Histone H2AX phosphorylation on serine residues is a hallmark for DNA double-strand breaks (DSBs), and its detection may serve as a sensitive marker to develop anticancer drugs [54].

Perturbation of the cell cycle and halting DNA replication may provide cells more time for DNA damage repair and prevent the incorporation of 5-FU metabolites into DNA, hence resulting in 5-FU resistance [55]. In accordance with the above phenomena, IFIT2-knockdown cells treated with 5-FU showed an accumulation of cells in S phase and less DNA damage than sensitive sh-control cells. Similarly, the S phase arrest in cells resistant to 5-FU indicated high TS expression after 5-FU treatment in head and neck carcinoma cells [56]. 5-FU activates Chk1 and arrests cells at S phase, and its inhibition sensitizes tumor cells to 5-FU [57]. We also observed increased pChk1 expression in IFIT2-knockdown cells, supporting the view that halting DNA replication may prevent the misincorporation of 5-FU metabolites into DNA and prolong the doubling time for repairing 5-FU-induced DNA damage.

Accordingly, IFIT2 knockdown markedly prevented 5-FU-induced DNA damage. In general, 5-FU exerts its DNA damaging effects through anabolic actions by which 5-FU is enzymatically converted into active metabolites and incorporated into DNA and RNA of the dividing cells, leading to DNA and/or RNA damage [58]. TS, the target of the 5-FU metabolite FdUMP (by forming the ternary complex TS/CH_2_-THF/FdUMP), catalyzes the reaction of dUMP/FdUMP to deoxythymidine monophosphate (dTMP)/FdTMP [59]. Numerous reports have shown that increased TS expression is one of the common mechanisms concerning 5-FU resistance [52,56,58,60,61]. By examining the formation of the ternary complex (TS/CH_2_-THF/FdUMP), we observed relatively high levels of free TS in IFIT2-knockdown cells, confirming that their resistance to 5-FU is associated with the insufficient inhibition of TS [59]. An acute induction of TS protein and enzyme activity in cells after 5-FU treatment is due to intracellular accumulation of nucleotides FdUMP and dUMP that competitively bind to TS, leading to inhibition of subsequent binding to TS mRNA and hence inhibiting the feedback suppression of TS on its expression. An increased intracellular level of free TS is more important than the bound (TS+5FU) form, as free TS is responsible for maintaining the thymidylate levels and DNA biosynthesis in cells [62]. The underlying regulatory mechanism of enhanced TS expression in IFIT2-depleted cells is still unclear and warrants our further investigation.

We also observed a significant increase in TK1 in IFIT2-knockdown cells. TK1 is involved in the salvage pathway, producing dTMP from deoxythymidine; dTMP is further phosphorylated to form deoxythymidine triphosphate (dTTP), which is essential for DNA replication and repair. However, the involvement of TK1 in 5-FU resistance remains controversial [63,64]. By aid of the siRNA technique, the silencing of TS alone or the double silencing of TS and TK1 but not TK1 alone sensitized IFIT2-knockdown cells to 5-FU. Taken together, our findings suggest that increased TS expression is likely responsible for 5-FU resistance in IFIT2-knockdown cells.

How to overcome 5-FU resistance is a clinical challenge. SAHA overcomes 5-FU resistance by downregulating TS expression by blocking the Rb-E2F1 pathway in lung cancer cells [46]. The synergistic interaction between SAHA and 5-FU in both double and triple combinations with different therapeutic drugs in different cancer cell types has previously been reported to enhance the effects of 5-FU by increasing apoptosis and decreasing proliferation [47]. The synergistic interaction has been shown to modulate the expression of the metabolic enzymes TS and TP, which are involved in the mechanism of resistance to fluoropyrimidines [46,65,66,67,68]. Our results also support this hypothesis, as SAHA treatment led to the downregulation of TS protein expression in a dose-dependent manner. In addition, a low dose of SAHA in combination with 5-FU enhanced sensitivity in IFIT2-knockdown cells and effectively suppressed their growth in mouse xenografts. Reported evidence has shown that TS expression is regulated by multiple molecular mechanisms, including transcriptional activity, mRNA stability and protein stability. HDAC inhibitors have been reported to induce the transcriptional repression of TS mRNA as well as destabilize the TS protein. HDAC inhibitors were also reported to induce Hsp90 acetylation, which resulted in decreased chaperone activity and destabilized the TS protein [60]. We therefore hypothesize that HDACs may regulate the expression and stability of the TS protein, leading inherently to 5-FU resistance in IFIT2-depleted cells.

Drug resistance, metastasis and recurrence have been the major hurdles for oral cancer treatment. Therefore, it is essential to elucidate the crucial molecular pathways associated with drug resistance and recurrence in OSCC and to identify a novel biomarker to develop more effective treatments and improve patient survival. The treatment of OSCC, particularly recurrent disease due to drug resistance, is very challenging. Therefore, it is crucial to understand the molecular mechanisms of drugs under development as well as drugs on clinical use so that such drugs can be used to improve current clinical practices to circumvent resistance and metastasis. In addition, TS expression may serve as a predictor of the clinical response to 5-FU therapy [61], implicating that IFIT2 expression may also be used as a biomarker for 5-FU treatment. Whether patients with reduced IFIT2 expression might show poor 5-FU efficacy and develop resistance warrants further investigation. Moreover, SAHA combined with 5-FU has the potential to enhance the efficacy of 5-FU-based therapy by overcoming TS-mediated drug resistance and opening a new therapeutic option for OSCC.

## 4. Materials and Methods

### 4.1. Cell Lines and Cell Culture

sh-IFIT2-1, sh-IFIT2-2 and sh-control cells derived from the human OSCC cell line CAL27 were generated earlier as previously described [16] and maintained in DMEM supplemented with 10% fetal bovine serum, 100 units/mL penicillin, 100 μg/mL streptomycin and 0.2 µg/ml puromycin (Sigma-Aldrich, St. Louis, MO, USA). Cells were incubated in humidified conditions with 5% CO_2_ at 37 °C. All the culture medium and its supplements were purchased from Gibco Life Technologies (Rockville, MD, USA).

### 4.2. Cytotoxicity Assay

The cytotoxicity of various drugs in cells was assessed by seeding 3000 cells into each well of a 96-well plate, followed by incubation overnight and treatment with various concentrations of the drugs for 72 h. Cisplatin dichloride, mitoxantrone dichloride, raltitrexed, cytarabine and paclitaxel were purchased from Sigma; 5-FU was purchased from Fluka; docetaxel, gemcitabine and oxaliplatin were obtained from the USP; gefitinib and irinotecan were purchased from Caymann; and doxorubicin dihydrochloride was obtained from Pfizer. Cell growth was determined by the PrestoBlue (Invitrogen, Carlsbad, CA, USA) assay using a microplate spectrometer and fluorescence microplate spectrophotometer. By aid of CompuSyn software (version 1.0.1; CompuSyn, Inc., Paramus, NJ, USA), the IC_50_ value of each drug was calculated based on the median-effect principle and plotted using the dose–effect relationship at nine concentrations [69]. The same protocol and software were adopted to determine the combination index (CI) of the combination treatment.

### 4.3. Comet Assay

The comet assay was performed to evaluate DNA damage induced by 5-FU [70]. An aliquot of 8 × 10^5^ cells was seeded onto 6-well plates. After overnight incubation, the cells were treated with 10 µM 5-FU for 24, 48 and 72 h. At the end of treatment, cells were trypsinized, gently dispersed in low-melting agarose solution, embedded in 1% agarose on precoated microscope slides and immersed in lysis buffer (100 mM sodium EDTA, 2.5 mM NaCl and 10 mM Tris–HCl, pH 10) containing 1% Triton X-100 and 10% dimethyl sulfoxide (DMSO) overnight. Before electrophoresis, the slides were submerged in alkali buffer (300 mM NaOH, 1 mM sodium EDTA, pH 13.5) for 20 min. Electrophoresis was conducted at 25 V and 300 mA in the same buffer for 45 min. After electrophoresis, the slides were neutralized with neutralizing buffer (250 mM Tris–HCl, pH 7.5) overnight and then stained with 20 μM YOYO-1 dye (Molecular Probes, Eugene, OR, USA). The comet image was obtained under a fluorescence microscope (shortwave pass filter, 450–490 nm; chromatic beam splitter, 510 nm; and longwave pass filter, 520 nm) equipped with a digital camera (DCS-420; Kodak, Rochester, NY, USA). The tail moment of 100 cells for each treatment was analyzed using Comet Assay III software.

### 4.4. Western Blot Analysis

Western blot analysis was performed as previously described [16]. In general, cell lysates were extracted using RIPA buffer from Millipore. An aliquot of an equal amount of cell extracts (~50) μg was electrophoretically separated on sodium dodecyl sulfate-polyacrylamide gels and transferred onto polyvinylidene difluoride membranes (Amersham Bioscience Healthcare Bio-Sciences Corp., Piscataway, NJ, USA). Next, the membrane was subjected to incubation with the primary antibody (1:1000 dilution) overnight at 4 °C, followed by incubation with the horseradish peroxidase-conjugated anti-rabbit or anti-mouse secondary antibody (1:3000 dilution) for 1 h at room temperature. The specific protein bands were visualized by chemiluminescence using the Immobilon Western chemiluminescent HRP substrate reagent (EMD Millipore, Billerica, MA, USA). Primary anti-TS (sc-390945), anti-OPRT (sc-398086), anti-TP (sc-47702) and anti-TK1 (sc-377211) were obtained from Santa Cruz Biotechnology (Santa Cruz, CA, USA); anti-c-caspase 9 (#9509), anti-c-PARP (#5625), anti-Chk1 and anti-phospho-Chk1 S317 were obtained from Cell Signaling Technology (Danvers, MA, USA); anti-γH2AX was purchased from Upstate (NY, USA); and anti-GAPDH (#60004-1-Ig) was purchased from Proteintech (Chicago, IL, USA). Densitometry was performed using ImageJ software [71]. The uncropped images of Western blots are provided in the Figure S4.

### 4.5. Cell Cycle Analysis

The effects of 5-FU on cell cycle progression were analyzed by flow cytometry as previously described [72]. In brief, 2 × 10^5^ cells were seeded into 6-well plates and incubated at 37 °C overnight. At the end of treatment with 10 µM 5-FU for 24, 48 and 72 h, the attached cells were trypsinized, fixed in ice-cold 70% ethanol and maintained at −20 °C overnight. The cells were then stained with Hoechst 333,424 dye and subjected to flow cytometric analysis (FACScan flow cytometer; Becton Dickinson, San Jose, CA, USA). Cell cycle phase distribution was analyzed using FlowJo 10 software (Verity Software House, Topsham, ME, USA).

### 4.6. Annexin V Staining Assay

To analyze apoptotic cell death after 5-FU treatment, annexin staining was performed after 24, 48 and 72 h of 5-FU treatment by staining with annexin V-APC and PI (eBioscience, San Diego, CA, USA) according to the manufacturer’s instructions. The stained cells were analyzed by flow cytometry using an LSRII-17 color cytometer.

### 4.7. Quantitative Real-Time PCR (Q-PCR)

mRNA expression was determined by Q-PCR according to a previously described method [17]. Total RNA was extracted from cells using TRIzol reagent (Thermo Fisher Scientific, Waltham, MA, USA), and cDNA was synthesized using an iScript™ cDNA Synthesis Kit (BIO-RAD, Hercules, CA, USA). Q-PCR was performed using a Light Cycler 480 system (Roche Diagnostics, Pleasanton, CA, USA) with a final volume of 20 µL containing 5 µL of cDNA, 5 µL of SYBR Green I Master Mix (Roche Diagnostics, Indianapolis, IN, USA) and 200 nM of the appropriate primers. The relative differences in mRNA levels between genes were determined using cycle threshold (Ct) values as follows: the Ct value of the gene of interest was normalized to that of GAPDH in the same sample, and then the difference between groups was calculated and expressed as an increase or decrease in cycle numbers compared with the control. The primer sequences are listed in Table S1.

### 4.8. Immunofluorescence Staining

Immunofluorescence staining was performed to visualize the intracellular location of TS [2]. In brief, the cells cultured on slides were treated with or without 10 μM 5-FU for 72 h, washed with cold PBS and fixed with 100% ice-cold methanol for 30 min. Slides were then washed twice with PBS, permeabilized with PBS containing 0.2% Triton X-100 for 10 min and incubated overnight with the TS antibody at 4 °C. The next day, anti-mouse IgG Alexa Fluor^®^ 555 (Molecular Probes, Eugene, OR, USA) was added and incubated at 37 °C for 1 h. The nuclei were counterstained with 4′,6-diamidino-2-phenylindole (DAPI). After mounting with 50% glycerol in PBS, images were acquired with a laser scanning confocal microscope (LSM 700, Carl Zeiss Microlmaging Inc., Thornwood, NY, USA) and AxioVision software.

### 4.9. siRNA Transfection

Vector control cells (sh-control) and IFIT2-knockdown cells (sh-IFIT2-1 and sh-IFIT2-2 cells) were plated at a density of 3 × 10^5^ cells per well into 6-well culture dishes and incubated in humidified conditions with 5% CO_2_ at 37 °C. The next day, a siRNA smart pool from Dharmacon (Lafayette, CO, USA) was transfected into the cells using Lipofectamine RNAiMAX reagent (Invitrogen Life Technologies, Carlsbad, CA, USA) according to the manufacturer’s instructions. The ON-TARGET plus siRNA smart pool of four siRNAs used to target human TS and TK1 mRNAs and the negative control siRNA were obtained from Dharmacon.

### 4.10. Xenograft Animal Model

Animal experiments were performed according to the guidelines of the Institutional Animal Care and Utilization Committee of Academia Sinica. Xenograft animal models were established by subcutaneous injection of 1 × 10^7^ cells into the dorsal flank region of 5-week-old male mice (BALB/cAnN.Cg-Foxn1nu/CrlNarl) as previously described [2]. When the transplanted tumor reached approximately 100 mm^3^, nude mice were randomized into four groups (*n* = 4–5/group) and treated with the following drug regimens: (i) vehicle (10% DMSO/45% PEG300/45% saline) administered orally, (ii) SAHA (80 mg/kg) formulated in vehicle given orally 3 times for 4 consecutive days with a 3-day interval, (iii) 5-FU (100 mg/kg) given via i.p. in 100 µL of saline once per week for 3 weeks and (iv) SAHA combined with 5-FU. Tumor volumes and body weight were measured every other day. Tumor volume was measured along the longest orthogonal axes and calculated as follows: volume = 1/2 (length × width^2^), where width is the shortest measurement.

### 4.11. Statistical Analysis

GraphPad software (version 6.0) was used for data analysis. Comparisons of expression levels between two groups were performed using Student’s *t*-test to determine significant differences. *p*-values < 0.05 were considered statistically significant.

## 5. Conclusions

IFIT2 expression is associated with cell migration, metastasis, invasion, angiogenesis and chemoresistance. The underlying mechanism of all the above phenomena, except for drug resistance, involves aPKC signaling or TNFα. In this report, we show that IFIT2-depleted cell lines are resistant to 5-FU that can be circumvented by combining 5-FU with SAHA.

## Figures and Tables

**Table 1:** In vitro cell viability assay of different therapeutic agents in IFIT2-knockdown cells ^a^.

Therapeutic Agent	sh-Control	sh-IFIT2-1	sh-IFIT2-2
**ANTIMETABOLITE**			
5-Fluorouracil	3.03 ± 0.83	13.37 ± 1.66 (4.4) ^b,^***	11.08 ± 1.31 (3.7) ***
Cytarabine	0.67 ± 0.24	0.91 ± 0.13 (1.4) ***	1.19 ± 0.22 (1.8) ***
Gemcitabine	0.011 ± 0.000	0.018 ± 0.001 (1.7) **	0.014 ± 0.001 (1.3) *
Raltitrexed	0.105 ± 0.003	0.126 ± 0.001 (1.2) **	0.123 ± 0.002 (1.2) *
**TOPOISOMERASE INHIBITOR**			
Irinotecan (I)	3.88 ± 1.04	3.94 ± 0.55 (1.0)	4.02 ± 0.64 (1.0)
Doxorubicin (II)	0.08 ± 0.02	0.10 ± 0.02 (1.3) *	0.08 ± 0.01 (1.0)
Etoposide (II)	0.94 ± 0.35	1.04 ± 0.29 (1.1)	0.97 ± 0.38 (1.0)
Mitoxantrone (II)	0.003 ± 0.001	0.003 ± 0.000 (1.0)	0.003 ± 0.001 (1.0)
**PLATINUM BASED**			
Cisplatin	3.38 ± 0.80	4.15 ± 0.96 (1.2)	3.65 ± 1.17 (1.1)
Carboplatin	31.36 ± 0.99	34.45 ± 4.03 (1.1)	29.96 ± 7.67 (1.0)
Oxaliplatin	6.11 ± 2.58	10.36 ± 3.98 (1.7) **	8.53 ± 1.41 (1.4) ***
**TYROSINE KINASE INHIBITOR**			
Gefitinib	5.10 ± 1.2	8.27 ± 2.54 (1.6) *	9.74 ± 1.42 (1.9) **

^a^ Data (IC_50_) are shown as the mean ± SD (µM) of four to eight independent experiments. ^b^ Parentheses indicate the resistance factor (IC_50_ of IFIT2-knockdown cells/IC_50_ of sh-control cells). * *p* < 0.05, ** *p* < 0.01, and *** *p* < 0.001; compared with sh-control cells by Student’s *t*-test.

## Supplementary Materials

The following are available online at https://www.mdpi.com/2072-6694/12/12/3527/s1, Table S1: Primer Sequences for quantitative real time PCR analysis, Figure S1: Representative images of Comet assay, Figure S2: Representative histograms of cell-cycle analysis, Figure S3: Representative image of annexin V staining for apoptosis analysis using flow cytometry, Figure S4: Original images of Western.
