# Supplementary material for: SAHA Overcomes 5-FU Resistance in IFIT2-Depleted Oral Squamous Cell Carcinoma Cells
Source: Cancers (Basel). 2020 Nov 26;12(12):3527. doi: 10.3390/cancers12123527 (PMC7761248; doi:10.3390/cancers12123527)
Supplement: Supplementary file 1 [file cancers-12-03527-s001.pdf]

Supplementary Materials: SAHA Overcomes 5-FU Resistance in IFIT2-Depleted Oral Squamous Cell Carcinoma Cells

Prabha Regmi, Kuo-Chu Lai, Chung-Ji Liu and Te-Chang Lee

Table S1. Primer sequences for quantitative real-time PCR analysis.

Gene	Forward Primer	Reverse Primer
TS	5′-GGAAGGGTGTTTTGGAGGAGTT-3′	5′-AGATTTTCACTCCCTTGGAAGACA-3′
TP	5′-GCTGGAGTCTATTCCTGGAT-3′	5′-ACTGAGAATGGAGGCTGTGATG-3′
OPRT	5′-GCGTCTTCTGAGTCAGGTTG-3′	5′-GCATCTGCTAGCTGCAACAG-3′
TK1	5′-GTTTTTCCCTGACATCGTGGA-3′	5′-CGAGCCTCTTGGTATAGGCG-3′
GAPDH	5′-GGAGTCCCTGCCACACTCA-3′	5′-GCCCCTCCCCTCTTCAAG-3′

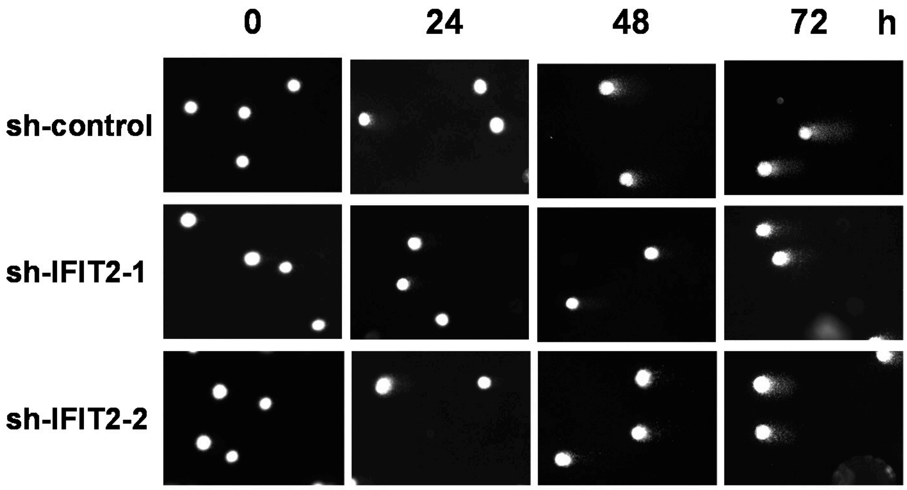

Figure S1. Representative images of Comet assay. Growing sh-control, sh-IFIT2-1 and sh-IFIT2-2 cells were treated with 10Mm 5FU for 12, 24, 48 and 72 h. At the end of treatment, the cells were harvested and subjected to a comet assay as described in the Materials and Methods.

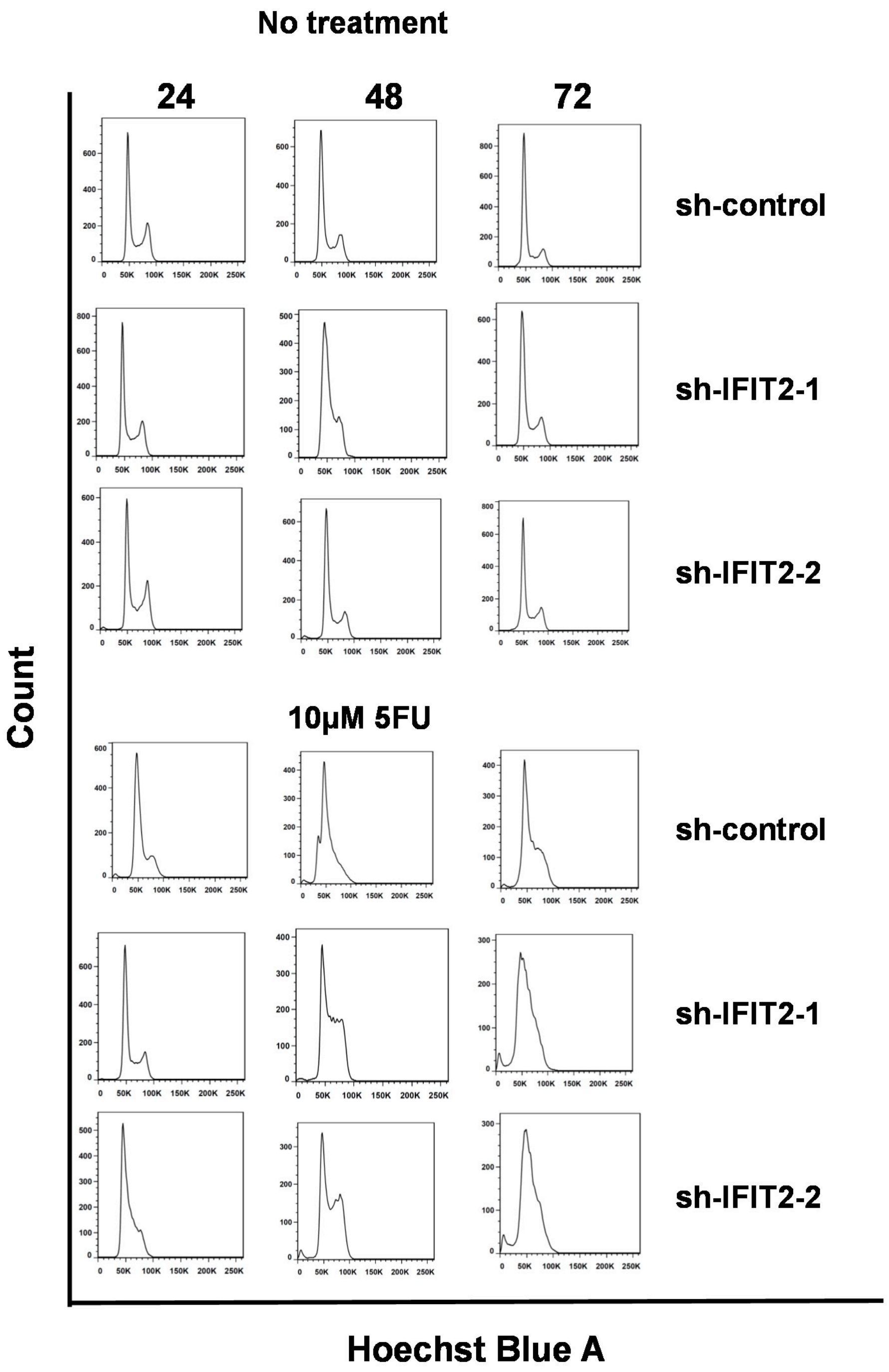

Figure S2. Representative histograms of cell-cycle analysis. Growing sh-control, sh-IFIT2-1 and sh-IFIT2-2 cells were treated with 10 µM 5-FU for 24, 48 and 72 h. and the cell-cycle analysis was performed using the Hoechst dye as described in methods.

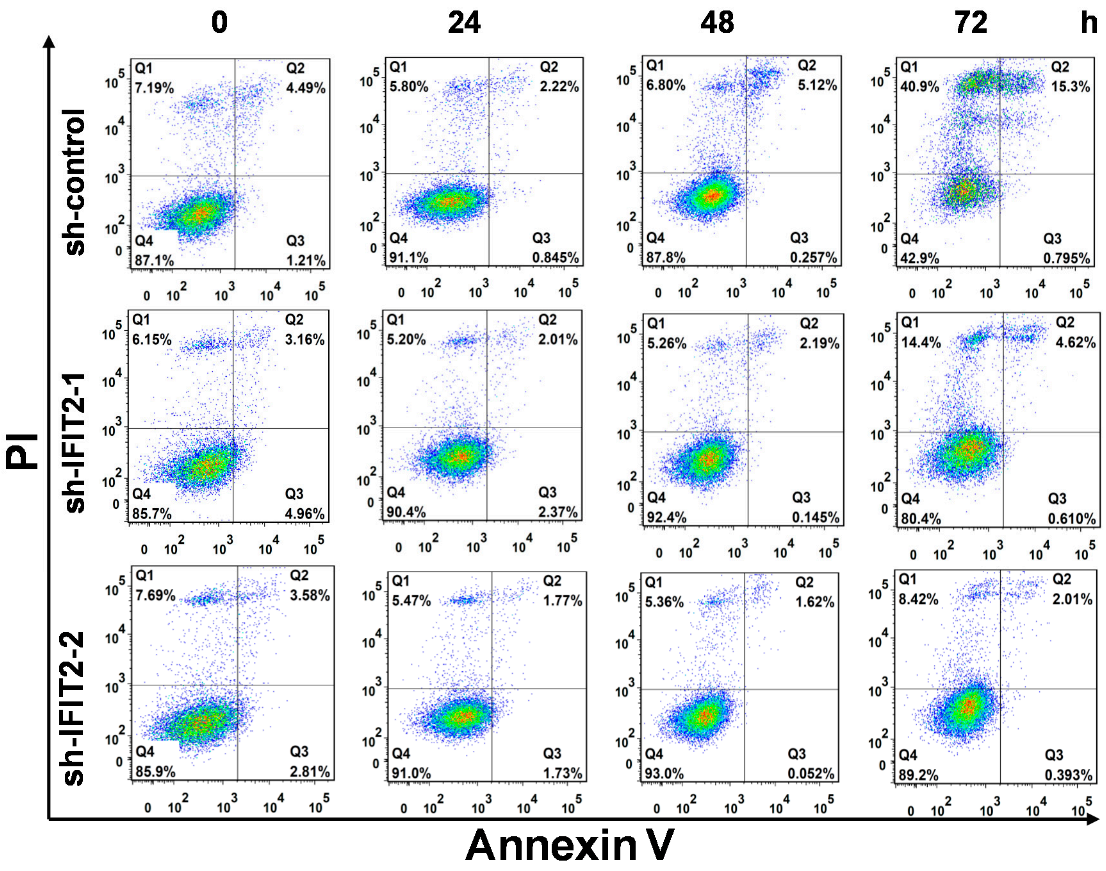

Figure S3. Representative image of annexin V staining for apoptosis analysis using flow cytometry. Cells were treated with 5-FU for 24, 48 and 72 h, stained with annexin V-APC and PI, and subjected to flow cytometric analysis**.** Q1, Q2, Q3, Q4 represent necrosis, late apoptosis, early apoptosis and live cells respectively.

Figure 1

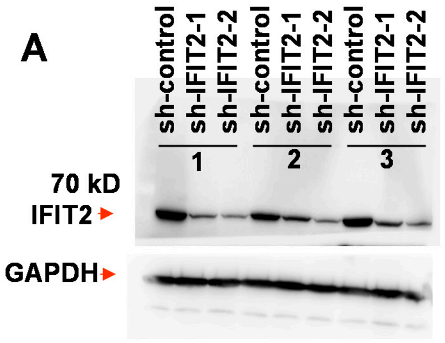

Membrane were cut to detect IFIT2 upper band and GAPDH lower band. The numbers 1,2,3 represent cell lysate of different passage number.

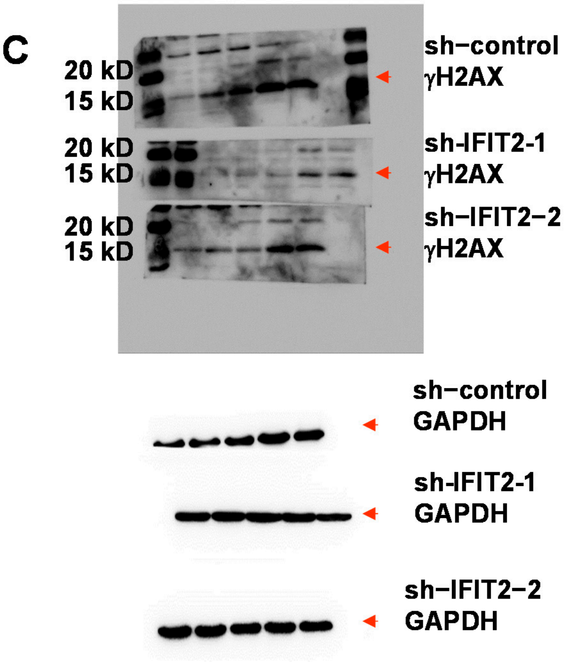

Figure 2B

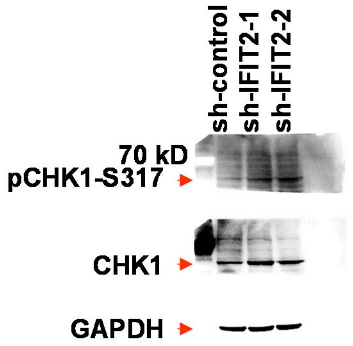

Figure 3C

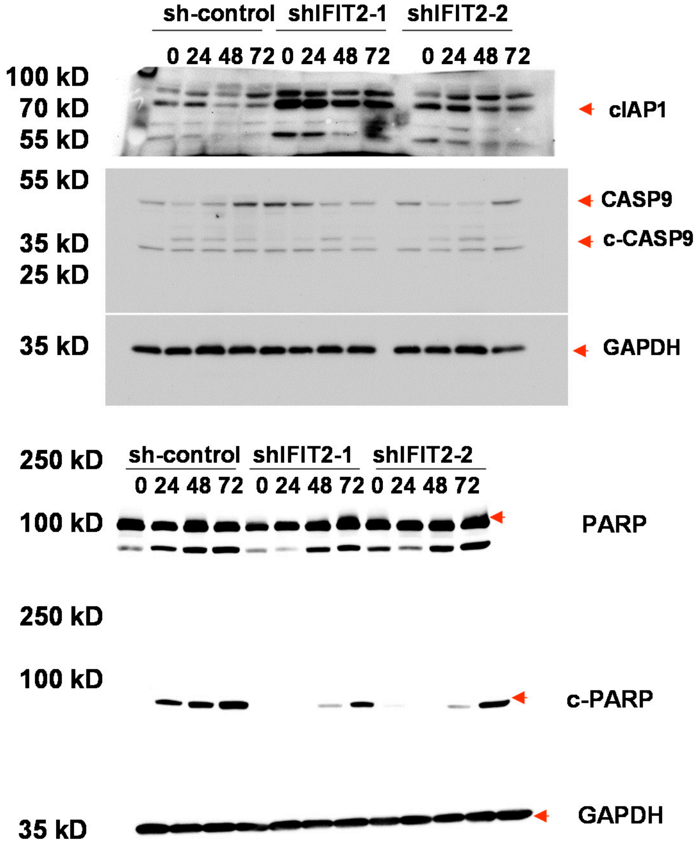

Figure 4

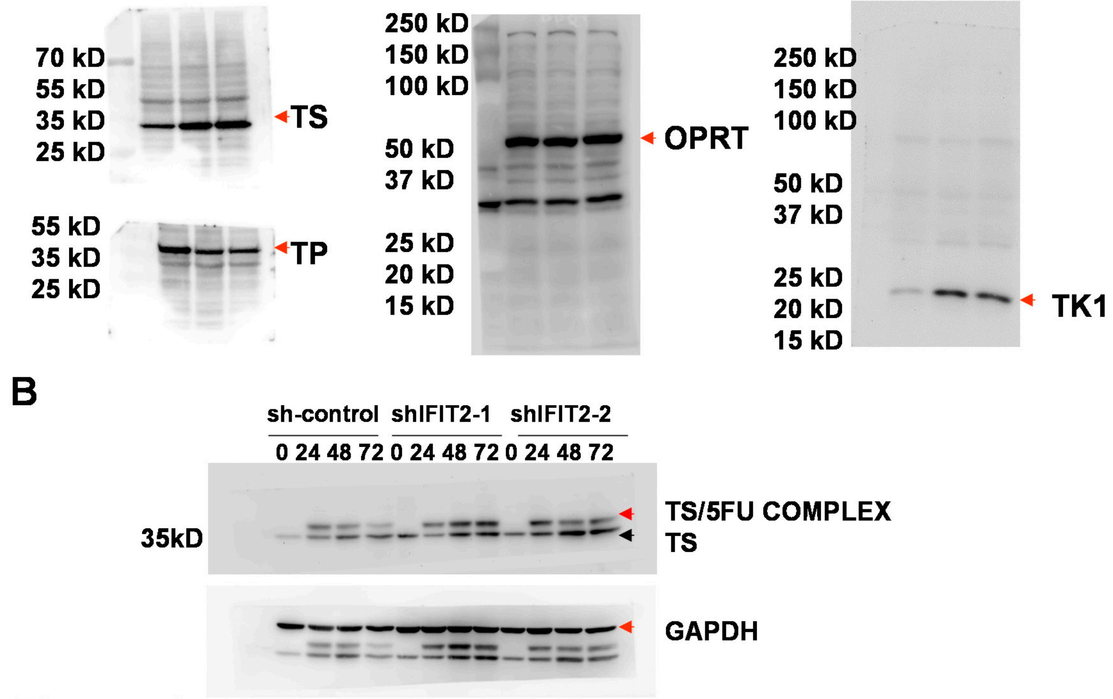

Figure 5A

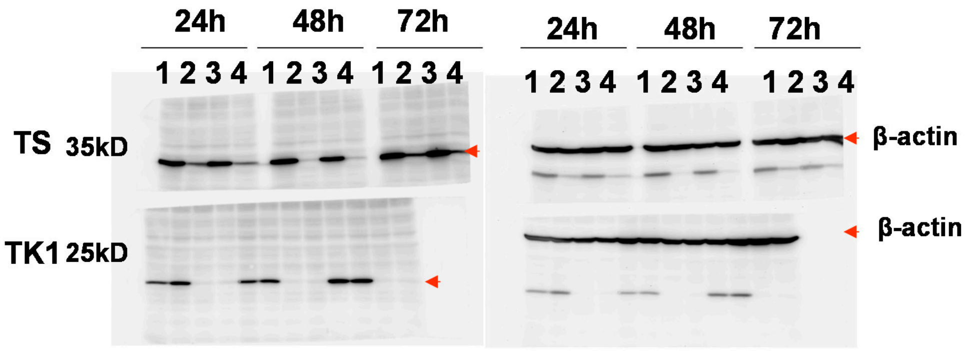

Label 1,2,3,4 represent different siRNA used as follows:
1. CONTROL siRNA
2. TS siRNA
3. TK1 siRNA
4.TS+TK1 siRNA
24h panel represents transfection efficiency after 24h

Figure 7A

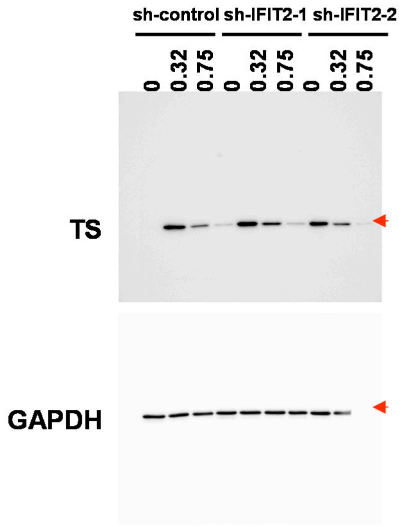

Figure S4. Original images of Western blots.

Publisher's Note: MDPI stays neutral with regard to jurisdictional claims in published maps and institutional affiliations.

© 2020 by the authors. Licensee MDPI, Basel, Switzerland. This article is an open access article distributed under the terms and conditions of the Creative Commons Attribution (CC BY) license (http://creativecommons.org/licenses/by/4.0/).
